# Evaluation of the Incidence of Human Papillomavirus–Associated Squamous Cell Carcinoma of the Sinonasal Tract Among US Adults

**DOI:** 10.1001/jamanetworkopen.2022.55971

**Published:** 2023-02-14

**Authors:** Nyall R. London, Melina J. Windon, Ameen Amanian, Fernando T. Zamuner, Justin Bishop, Carole Fakhry, Lisa M. Rooper

**Affiliations:** 1 Department of Otolaryngology–Head and Neck Surgery, Johns Hopkins University School of Medicine, Baltimore, Maryland; 2 Sinonasal and Skull Base Tumor Program, National Institute on Deafness and Other Communication Disorders, National Institutes of Health, Bethesda, Maryland; 3 Department of Neurosurgery, Johns Hopkins University School of Medicine, Baltimore, Maryland; 4 Department of Pathology, University of Texas Southwestern Medical Center, Dallas; 5 Department of Pathology, Johns Hopkins University School of Medicine, Baltimore, Maryland

## Abstract

This case series assesses the incidence of human papillomavirus (HPV)-associated sinonasal squamous cell carcinoma (SNSCC) and the prevalence of HPV-positive SNSCC among US adults.

## Introduction

Human papillomavirus (HPV) is responsible for a significant rise in oropharyngeal squamous cell carcinoma (OPSCC) incidence in the US, accounting for approximately 80% of OPSCCs.^1^ However, this epidemiologic trend has not been reported outside the oropharynx. HPV-associated sinonasal squamous cell carcinoma (SNSCC) predominantly arises from the nasal cavity and ethmoid sinuses, affects younger patients, and has an improved survival rate (albeit to a lesser degree than OPSCC).^2,3,4^ We hypothesized that the incidence and prevalence of HPV-associated SNSCC may be increasing over time.

## Methods

This study followed the reporting guideline for case series in medicine. Eligibility criteria and additional details are provided in the eMethods in Supplement 1.

The National Cancer Institute has strongly discouraged the use of HPV status as reported in the Surveillance, Epidemiology, and End Results Program (SEER) database to estimate HPV-positive incidence or trends due to bias and variability in the proportion of cases with unknown HPV status.^5^ Analogous to other robust studies that have evaluated epidemiologic trends,^6^ we used anatomic sites with greater proportion of HPV-positive tumors as a proxy for HPV-associated SNSCC.^4^

Data were analyzed from January 1, 1995, to December 31, 2019. For SEER analysis, the joinpoint regression model was used to calculate the annual percentage change (APC) in age-adjusted incidence rates. A 2-sample Wilcoxon rank-sum test was used to test for differences in patient age by HPV tumor status; *P* < .05 was deemed statistically significant. Analyses were performed using Stata, version 15.1 (StataCorp LLC). Additional details regarding statistical analysis are provided in the eMethods in Supplement 1.

## Results

**Figure 1.**
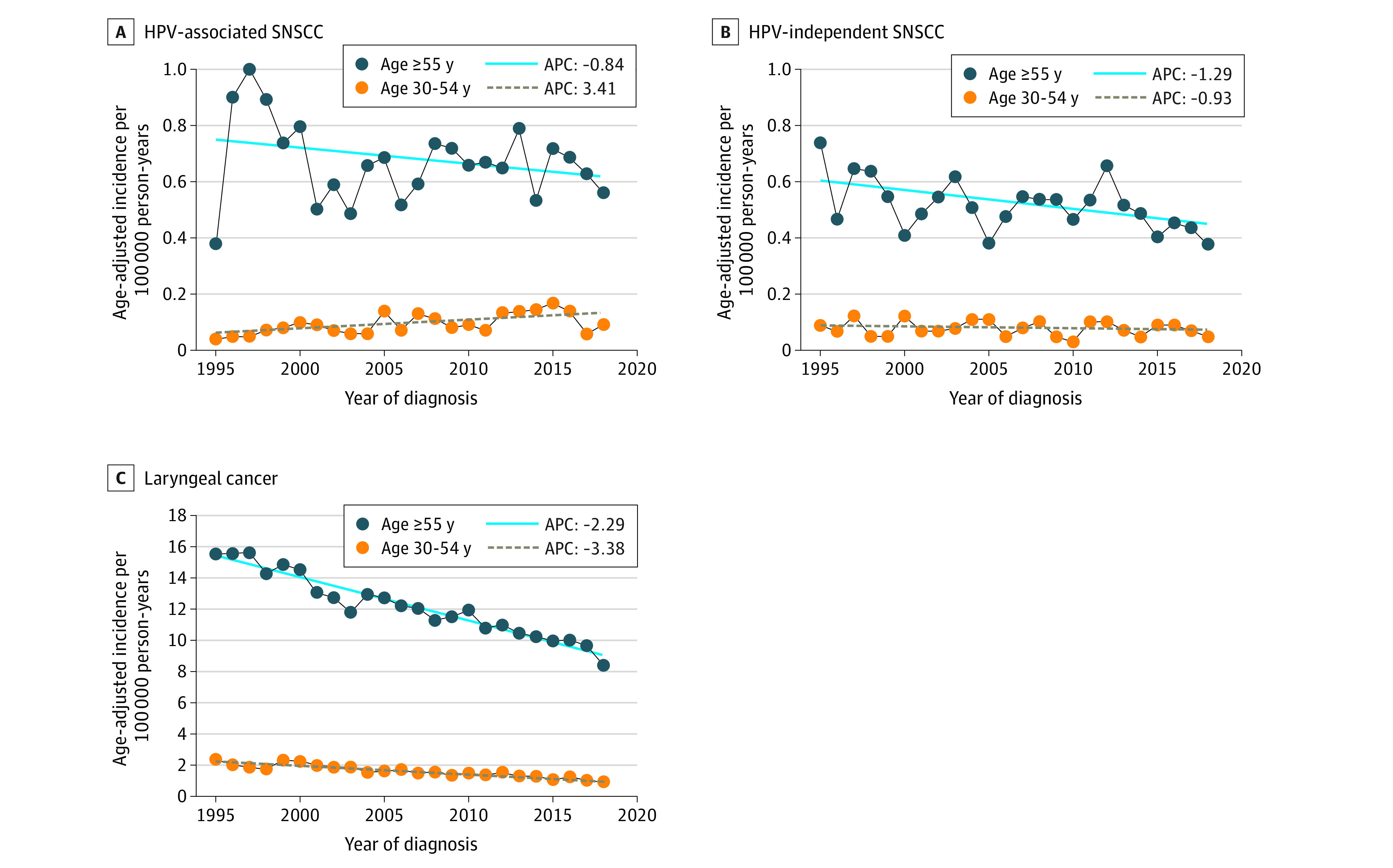
Age-Adjusted Incidence Trends By Calendar Year of Diagnosis (1995 to 2018) Stratified By Age at Diagnosis A, Human papillomavirus (HPV)-associated sinonasal squamous cell carcinoma (SNSCC) (nasal cavity and ethmoid sinus). B, HPV-independent SNSCC (maxillary, frontal, sphenoid, overlapping lesion of accessory sinuses, and accessory sinus not otherwise specified). C, Laryngeal cancer (HPV-independent). APC indicates annual percentage change.

We first assessed the incidence of HPV-associated SNSCC in the US population from 1995 to 2018. A significant increase in SNSCC at HPV-associated sites (APC, 3.41%; *P* = .003) was observed among individuals aged 30 to 54 years but not among individuals aged 55 years or older (Figure 1). In contrast, HPV-independent SNSCC incidence was stable among younger individuals and decreased significantly among older individuals (APC, −1.29%; *P* = .008) (Figure 1). The incidence of laryngeal cancer, a well-established HPV-independent head and neck cancer, decreased significantly for both younger (APC, −3.38%; *P* < .001) and older (APC, −2.29%; *P* < .001) age groups (Figure 1).

**Figure 2.**
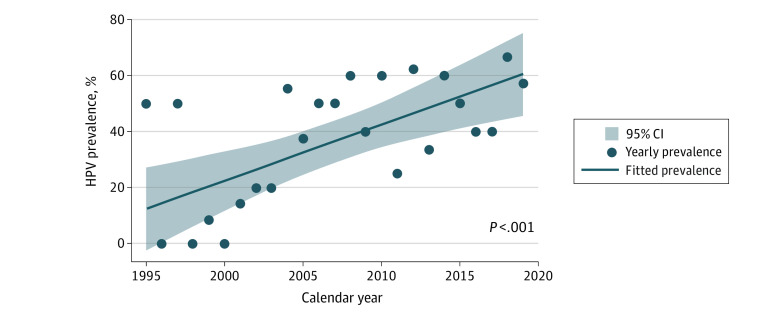
Prevalence of Human Papillomavirus (HPV)-Positive Sinonasal Squamous Cell Carcinoma High-risk HPV in situ hybridization of sinonasal squamous cell carcinoma samples from 1995 to 2019 demonstrated a significant 2.1% annual increase in prevalence of HPV-positive tumors over time (*P* < .001) after adjustment for age, similar to the unadjusted incidence.

In the institutional cohort, 62% of HPV-associated SNSCCs and 68% of HPV-independent SNSCCs were reported in male patients. Patients with HPV-associated SNSCC were significantly younger (median age, 56 y [IQR, 48-65 y]) than HPV-independent (median age, 63 y [IQR, 54-71 y]) (*P* = .001). Age-adjusted prevalence trends of HPV-positive tumor status among SNSCCs from 1995 to 2019 demonstrated a 2.1% (95% CI, 1.1%-3.1%) increase per year (*P* < .001) (Figure 2). The prevalence rose from 15% in 1995-1999 to around half of cases in later calendar periods (Figure 2). An increased odds of HPV-positive tumor status was observed for 2005 to 2009 (odds ratio [OR], 4.98 [95% CI, 1.36-18.21]), 2010 to 2014 (OR, 6.23 [95% CI, 1.66-23.32]), and 2015 to 2019 (OR, 5.41 [95% CI, 1.53-19.12]) when using 1995 to 1999 as the reference period. HPV16 was the predominant subtype in 37 of 54 HPV-associated SNSCCs (68.5%); among these, 36 (97.3%) were positive for p16. Of 96 HPV-negative tumors, 9 (9.4%) were positive for p16.

## Discussion

This study found an increase in the incidence of HPV-associated SNSCC and prevalence of HPV-positive SNSCC over recent decades. To our knowledge, this is the first study to suggest that the incidence of HPV-associated carcinoma outside the oropharynx is increasing. Collectively, these findings have important public health and clinical implications.

This study’s limitations include its retrospective design. The population-based approach found an increase in age-adjusted incidence rates in the nasal cavity and ethmoid sinus in younger patients, who are more likely to be HPV positive.^4^ Using anatomic subsites as a surrogate for HPV status may result in misclassification and may therefore underestimate the incidence of HPV-associated cases on a population level. This should be followed up by prospective, multi-institutional studies using high-risk HPV in situ hybridization.

## Notes

Supplement 1.
eMethods.

eReferences

Supplement 2.
Data Sharing Statement
