# Supplementary material for: Evaluation of the Incidence of Human Papillomavirus–Associated Squamous Cell Carcinoma of the Sinonasal Tract Among US Adults
Source: JAMA Netw Open. 2023 Feb 14;6(2):e2255971. doi: 10.1001/jamanetworkopen.2022.55971 (PMC9929694; doi:10.1001/jamanetworkopen.2022.55971)
Supplement: Supplement 1. — eMethods. eReferences [file jamanetwopen-e2255971-s001.pdf]

Supplemental Online Content

London NR Jr, Windon MJ, Amanian A, et al. Evaluation of the incidence of human papillomavirus–associated squamous cell carcinoma of the sinonasal tract among US adults. *JAMA Netw Open*. 2023;6(2):e2255971. doi:10.1001/jamanetworkopen.2022.55971

eMethods.

eReferences

This supplemental material has been provided by the authors to give readers additional information about their work.

© 2023 London NR Jr et al. *JAMA Network Open.*

eMethods

Study Design

This study consisted of two datasets. To explore incidence, patient demographic and cancer incidence data were extracted from the SEER database highlighting nine cancer registries from 1995-2018. To evaluate trends in SNSCC prevalence, HPV status was determined in a retrospective series of 151 consecutive cases of SNSCC identified at Johns Hopkins Hospital (Baltimore, MD) over a 25-year time period (1995-2019). This study was approved by the Johns Hopkins University Institutional Review Board.

HPV Status

In the SEER analysis, anatomic site was used to classify HPV-related and -unrelated sites. Given the higher proportion of HPV-related tumors in the nasal cavity and ethmoid sinus compared to other sinonasal subsites[1], cases with tumors from the nasal cavity (C30.0) and ethmoid sinus (C31.1) were considered HPV-related SNSCC (n=1245), whereas HPV-independent SNSCC (n=966) included the maxillary sinus (C31.0), frontal sinus (C31.2), sphenoid sinus (C31.3), overlapping lesion of the accessory sinus (C31.8), and accessory sinus NOS (C31.9). Laryngeal cancer sites (n = 22078) including glottis (C32.0), supraglottis (C32.1), subglottis (C32.2), laryngeal cartilage (C32.3), overlapping lesion of larynx (C32.8), and larynx, NOS (not otherwise specified) (C32.9), which are established to be HPV-independent were used an additional comparator cohort. Eligibility was restricted to squamous cell histologies (ICD-0-3: 8050-8076, 8078, 8083, 8084, 8094). SNSCC were classified according to the Internal Classification of Disease for Oncology version-3 [ICD-0-3].

© 2023 London NR Jr et al. *JAMA Network Open.*

In the single-institution analysis, immunohistochemistry for p16 (clone E6H4; Ventana Medical Systems, Tucson, AZ) was performed on all cases. P16-positivity was defined as strong, diffuse nuclear and cytoplasmic positivity in ≥70% of tumor cells as specified by College of American Pathologists Guidelines[2]. HPV-specific testing was performed via RNAscope RNA in situ hybridization (ISH, (Advanced Cell Diagnostics, Hayward, CA)) using a cocktail probe recognizing 18 high-risk types (16, 18, 26, 31, 33, 35, 39, 45, 51, 52, 53, 56, 58, 59, 66, 68, 73, and 82). Cases that showed multiple nuclear and/or cytoplasmic signals that were visible at least 200x magnification were deemed HPV-related[3]. HPV16 type-specific ISH was also performed using a HPV16-specific RNAscope probe (Advanced Cell Diagnostics, Hayward, CA). Of note, HPV16 staining was not available on one sample due to exhaustion of tumor tissue on the tissue microarray. Cases and HPV/p16 results from 1995-2011 previously reported by our institution were included in this study[4]. All histologic interpretation was performed by head and neck pathologist (LR). Medical record abstraction was also performed on this cohort.

<u>Statistical Analysis</u>

SEER incidence rates were calculated per 100,000 person-years and age-adjusted according to the 2000 US Standard Population. Specifically, the calculated age-adjusted incidence rates were age-stratified into two groups: 30-54 and 55+. Trends in age-adjusted incidence rates over the years of cancer diagnosis were described as the annual percentage change (APC)[5]. The APC was calculated via the joinpoint regression model where several lines are connected via joinpoints to explain time trends over time. Up to three joinpoints were allowed to determine the optimal combination of joinpoints for the non-linear model. Statistical significance was assessed using the Monte Carlo Permutation method and $p < 0.05$ was deemed statistically significant[5]. The APC calculation was performed using the Joinpoint Regression Program (Version 4.9.1.0). In the

© 2023 London NR Jr et al. *JAMA Network Open.*

Johns Hopkins cohort, the prevalence of HPV-positive SNSCC among all SNSCC over calendar year and consecutive 5-year period was evaluated by linear and logistic regression, with statistical significance considered $p < 0.05$. Annual percentage change in this cohort was defined as the change in prevalence of HPV-positive tumors per year. As those with HPV-positive tumors tended to be younger, the APC was adjusted by patient age. Two-sample Wilcoxon rank-sum was used to test for differences in patient age by HPV tumor status. STATA 15.1 was used for statistical analysis.

© 2023 London NR Jr et al. *JAMA Network Open.*

eReferences

1. Chang Sing Pang KJW, Mur T, Collins L, Rao SR, Faden DL. Human Papillomavirus in Sinonasal Squamous Cell Carcinoma: A Systematic Review and Meta-Analysis. *Cancers (Basel).* 2020;13(1).
2. Fakhry C, Lacchetti C, Rooper LM, et al. Human Papillomavirus Testing in Head and Neck Carcinomas: ASCO Clinical Practice Guideline Endorsement of the College of American Pathologists Guideline. *J Clin Oncol.* 2018;36(31):3152-3161.
3. Bishop JA, Ma XJ, Wang H, et al. Detection of transcriptionally active high-risk HPV in patients with head and neck squamous cell carcinoma as visualized by a novel E6/E7 mRNA in situ hybridization method. *Am J Surg Pathol.* 2012;36(12):1874-1882.
4. Bishop JA, Guo TW, Smith DF, et al. Human papillomavirus-related carcinomas of the sinonasal tract. *Am J Surg Pathol.* 2013;37(2):185-192.
5. Kim HJ, Fay MP, Feuer EJ, Midthune DN. Permutation tests for joinpoint regression with applications to cancer rates. *Stat Med.* 2000;19(3):335-351.

© 2023 London NR Jr et al. *JAMA Network Open.*
