# Supplementary material for: Evaluation of the Incidence of Human Papillomavirus–Associated Squamous Cell Carcinoma of the Sinonasal Tract Among US Adults
Source: JAMA Netw Open. 2023 Feb 14;6(2):e2255971. doi: 10.1001/jamanetworkopen.2022.55971 (PMC9929694; doi:10.1001/jamanetworkopen.2022.55971)
Supplement: Supplement 2. — Data Sharing Statement [file jamanetwopen-e2255971-s002.pdf]

Data Sharing Statement

London, Jr. Evaluation of the Incidence of Human Papillomavirus-Associated Squamous Cell Carcinoma of the Sinonasal Tract Among US Adults. *JAMA Netw Open*. Published February 14, 2023. doi:10.1001/jamanetworkopen.2022.55971

Data
Data available: Yes
Data types: Deidentified participant data
How to access data: Please e-mail Nyall London at nlondon2@jhmi.edu regarding data requests
When available: With publication

Supporting Documents
Document types: None

Additional Information
Who can access the data: Researchers whose proposed use of the data has been approved
Types of analyses: For any purpose
Mechanisms of data availability: With signed data use agreement and institutional approval
